# Development and preliminary validation of a public health emergency competency model for medical staffs of national health emergency teams in China

**DOI:** 10.1186/s12913-022-08361-z

**Published:** 2022-08-13

**Authors:** Yuhan Zhao, Yuanyuan Wang, Ting Zhang, Miaomiao Wang, Xiaojun Ye, Xintian Wang, Hongwei Sun

**Affiliations:** 1 Department of Teacher Education, Weifang University, Weifang, 261600 Shandong Province China; 2 School of Population Medicine and Public Health, Peking Union Medical College, Beijing, 10010 China; 3 Department of Psychology, Weifang Medical University, Fuyanshan Campus, No. 7166, West Baotong Street, Weicheng District, Weifang, 261053 Shandong China

**Keywords:** Competency model, Public health emergency, Emergency medical team, Medical workers

## Abstract

**Background:**

In the present study, we attempted to develop and validate a participatory competency model for medical workers and then evaluate the current status of competency characteristics of Chinese medical workers.

**Methods:**

The competency model was constructed in a multistage process, including literature review, expert consultation, critical incident and focus group interview. A pilot study was conducted to refine the initial model among 90 participators and the viability and reliability were evaluated by a questionnaire survey among 121 medical workers. Then, the current status of competency characteristics was measured based on the final version of competency model.

**Results:**

In the pilot study, ten questionnaires were dropped for the poor quality and thus the eligible rate was 92% (138/150). KMO value was 0.785 and Bartlett test showed that the χ^2^ = 6464.546 (df = 903) and *p* value < 0.001. Then**,** 10 items with double loading and factor loading < 0.4 were deleted. Finally, 33 items were retained with the lowest factor loading value of 0.465. The validity and reliability of competency model were determined with Cronbach’s α coefficient of 0.975 and ICC value of 0.933. Finally, a revised competency model with 5 dimensions and 31 items was obtained. The overall competencies of current medical workers were in a high level, except for emergency knowledge related competencies. Age was an independent factor affecting the competencies.

**Conclusions:**

Our competency model was a reliable and validated tool for assessing the competences of medical staffs against public health emergencies, and the overall competencies of current medical workers in China were in a high level, except for emergency knowledge related competencies.

**Supplementary Information:**

The online version contains supplementary material available at 10.1186/s12913-022-08361-z.

## Introduction

In the past decade, there is an increasing number of public health emergencies, which has been a challenge for government agencies and public health agencies to prepare and response to the successive public health emergencies in China and abroad. In the early years of twenty-first century, the outbreak of the epidemic of severe acute respiratory syndrome (SARS) and the occurrence of disastrous event of 9/11 attack have been the early landmark events of health emergency [1]. Then, the natural disaster of 2010 earthquake in Haiti has caused 225,000 deaths and a large number of homeless [2]. The recent event of the novel coronavirus (COVID-19) outbreak continues to be the international public health concern, for its rapid transmission and lethality [3]. Thus, it is imperative to improve the ability of governments to address the public health issues.

An emerging number of emergency medical teams has been deployed in different countries all over the world, with the aim to provide assistance for affected regions against disasters [4]. For the increasing frequency and complex of public health emergencies, an emerging interest has been attracted in the improvement of Emergency Medical Service System (EMSS) in China, since 2003 SARS. Concerns with regard to the shortage of basic capacities and coordination of emergency medical teams have been raised. Previous evidence determined the insufficiency of emergency preparedness capability of medical teams in China [5, 6]. Thus, there is an urgent need of approaches to evaluate the quality of emergency medical teams and improve preparedness and response of public health emergency.

Currently, there is a lack of an approved standard for guiding education and training of members in emergency medical teams to improve the quality [7]. Competency based models have been recommended [8, 9]. In the present study, we developed and validated a competency model for the emergency medical teams. It is anticipated that it is helpful in guiding the education and training of emergency medical teams in China.

## Methods

### Scale development

**Fig. 1:**
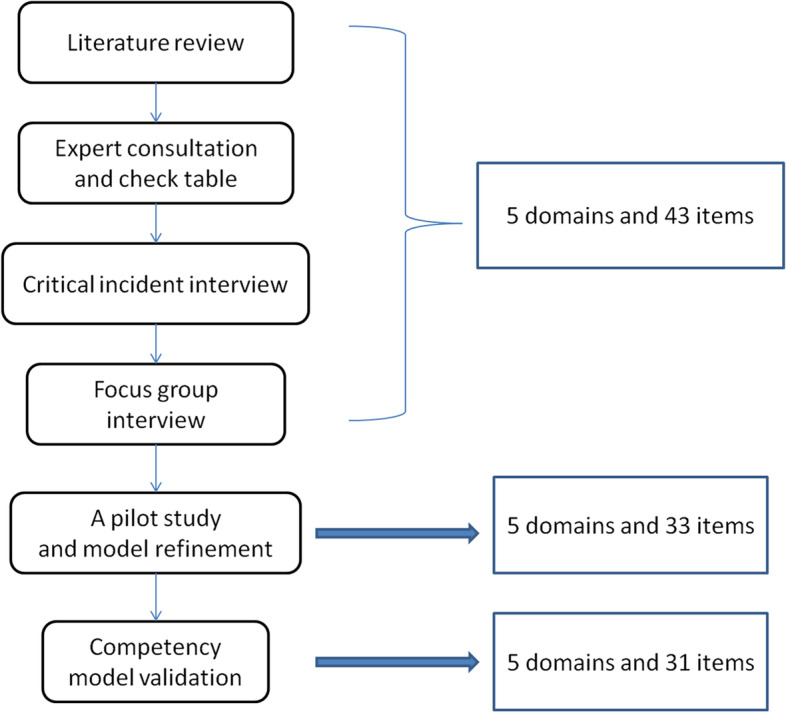
Flow chart of competency model development

The competency model for medical staffs was developed in a multistage process. The flow chart of competency model development was depicted in Fig. 1. The literature review was performed to search the review literatures about the competencies of health emergency team. In addition, a target internet search was performed for the internationally approved Competency Dictionary scaled by HayMcBer company and American CDC’s health emergency personnel competency scale in 2002. Based on the content review of the literatures and panel discussion, we compiled a check table of health emergency team member competencies, which contained 58 competencies.

This study was approved by the ethics committee of Weifang University (No.2021001), and informed consent has been signed by all participants.

### Item identification

Competences refer to individual potential traits such as knowledge, skills, ability, values, personality and motivation that lead to high management performance [10], and the competency elements in this study were identified by 3 kinds of interviews. First, the expert consultation was conducted with 3 experts (2 males and 1 female) from National Centers for Disease Control and Prevention. After consultation, the experts were invited to complete the check table of health emergency team member competencies. And the experts were invited according to the following inclusion criteria: (I) leaders in national health emergency team; or (II) leaders in provincial health emergency team; or (III) main leaders in national health emergency related units. Secondly, 5 members with excellent performance (4 males and 1 female) and 5 with common performance (3 males and 2 female) in National CDC Health Emergency Response Team were invited for the critical incident interview. Finally, the items were identified by 3 rounds of focus group interview, in which 5 experts (3 males and 2 females) were interviewed. The inclusion criteria for members with excellent performance contained (I) ≥ 10 years of work experience for health emergency response units; (II) professional field of emergency management, or preventive medicine, or clinical medicine and or rescue medicine; (III) associate senior or above (associate professor or associate researcher) working experience; (IV) participated in ≥ 15 health emergency responses.

The contents of interviews were digitally recorded and transformed to code format by 5 interview coding members. The results of interviews were merged into 5 domains with coding consistency coefficient of 0.73. According to the domains, competency items were modified or dropped. Finally, the initial competency model for national health emergency team was developed with 5 domains and 43 items.

### Model refinement

In August 25–31, 2019, there was a joint imported epidemic exercise in Kashgar, Xinjiang, China, which contained 90 participators from 9 regional CDC health emergency response teams. In addition, 60 participators in national disease control and health emergency team were recruited according to the inclusion criteria: (I) professional field of emergency management, or preventive medicine, or clinical medicine and or rescue medicine; (II) the non-support worker. For the importance of items assessment and self-evaluation of the initial version of our competency model, a pilot study was conducted with 150 questionnaires. The questionnaires with half-hearted answer and more than 50% of missing items were dropped. The responses of questionnaires were scored based on 5-point Likert scale, ranged from 1–5 points for each question. 1 represented strongly agree, while 5 indicated strongly disagree. Among 43 questions, one question (t18) was recorded by reverse scoring. The structural validity of scale was evaluated by Kaiser–Meyer–Olkin (KMO) test and Barlett test. When KMO measure is close to 1, it means there are common factors among the variables. *P* < 0.05 was set as the significant level in Barlett test, which indicated high correlations between factors.

### Structural validity and reliability analysis of the model

In 2020, a questionnaire survey based on the modified competency model was performed. Total 121 medical workers of 8 health emergency teams from 4 districts (Shandong, Xinjiang, Sichuan, Shanghai) were included. The self-evaluation of participators were rated by 5-point Likert scale according the procedure mentioned above. The internal consistency reliability was analyzed with Cronbach’s α ≥ 0.7 as significant. The test–retest reliability was estimated by Intraclass correlation coefficient (ICC). ICC represents excellent, when it is > 0.75, fair to good with value > 0.4 and poor with value < 0.4 [11]. Confirmatory factor analysis was performed to assess the goodness of fit for the competency model. A acceptable model fit was considered with comparative fit index (CFI), goodness-of-fit index (GFI) and incremental fit index (IFI) > 0.9. Additionally, the root mean square error of approximation (RMSEA) was calculated. RMSEA < 0.5 reflected a close fit, 0.05 < RMSEA < 0.08 indicated an acceptable fit and 0.08 < RMSEA < 0.1 remained to be acceptable, while RMSEA > 0.1 meant a poor fit.

### Evaluation of the current status of competence characteristics for emergency medical workers in China

To assess the current status of the competences of medical workers in response to the prevention and control of COVID-19 epidemic, a questionnaire survey based on the final version of competency model was conducted. Total 270 subjects in national emergency medical teams were recruited in this trial, which were from 7 regions, including Eastern China, Central China, Northern China, Southwest China, Northwest China, Southern China, and Northeast China. There were total 270 questionnaires and 234 questionnaires were returned, of which there were 227 valid questionnaires. The questionnaires were filled by responders based on self-evaluation. Each item was rated by 5-point Likert scale as mentioned above. The validity and reliability of questionnaires were detected by Cronbach’s α and ICC, respectively.

### Statistical analysis

All the data were analyzed by SPSS20.0 or AMOS21.0 software. The item analysis and factor analysis were achieved by SPSS20.0 and AMOS21.0 software, respectively. The reliability analysis was performed by SPSS20.0. The continuous data were represented as mean ± standard deviation (SD) and the enumeration data were displayed by percentage. Two-group comparison was conducted by t test and the multi-group analysis was performed by multivariate analysis of variance (MANOVA).

## Results

### Competency model refinement

**Fig. 2:**
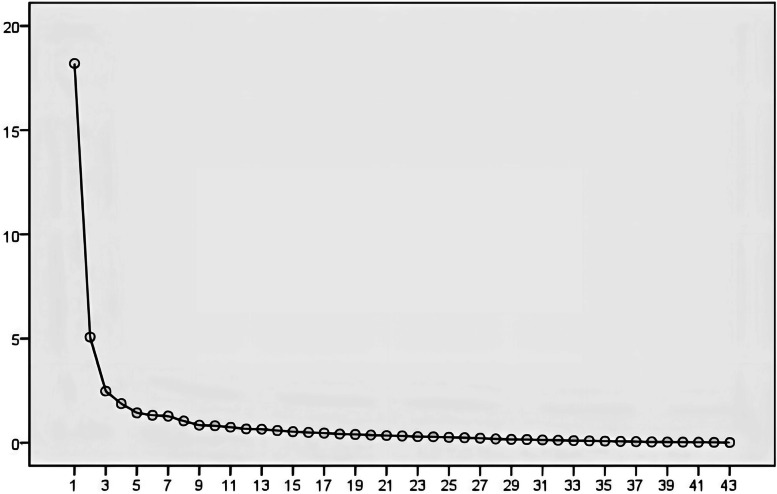
Factor analysis of the scree plot

In the pilot study, total 150 medical workers were invited to full fill the questionnaires according to initial version of competency model. The response rate was up to 98.67% (148/150). Ten questionnaires were dropped for the poor quality and the eligible rate was 92% (138/150). Based on the data of 148 questionnaires, KMO value was determined to be 0.785, close to 1, which indicated common factors among variables. Additionally, the results of Bartlett test showed that the χ^2^ value was 6464.546 (df = 903) and *p* value was < 0.001, suggesting that there were correlations between common factors. The results indicated that the scale was suitable for factor analysis. As shown in Fig. 2 and Table 1, factor analysis revealed that there were 5 domains and the cumulative percentage of variance of 2 domains was 79.32%. After several rounds of factor analysis, 10 items with double loading and factor loading < 0.4 were deleted. Finally, 33 items were retained with the lowest factor loading value of 0.465.

**Table 1:** Results of factor anlaysis in the questionaire survey of the pilot study

Question	Factor 1	Factor 2	Factor 3	Factor 4	Factor 5
t 1	0.607				
t 3	0.793				
t 4	0.668				
t 5	0.704				
t 7	0.831				
t 8	0.782				
t 9	0.767				
t 10	0.695				
t 13	0.675				
t 26		0.502			
t 31		0.737			
t 32		0.723			
t 33		0.746			
t 34		0.710			
t 35		0.680			
t 36		0.559			
t 37		0.675			
t 14			0.805		
t 15			0.666		
t 16			0.852		
t 17			0.693		
t 18			0.654		
t 19			0.706		
t 24			0.558		
t 27			0.465		
t 20				0.813	
t 21				0.801	
t 22				0.830	
t 23				0.769	
t 28					0.642
t 38					0.455
t 39					0.595
t 40					0.516

### Competency model validation

To assess the construct validity and reliability of competency model, a cohort of 121 subjects were surveyed with 33-item questionnaire. Total 118 questionnaires were responded, among which 110 ones were valid with the effective rate of 91%. The basic characteristics of the 118 respondents were depicted in Table 2. All the respondents aged 21 to 60 years, among which 60.2% aged 31 to 40 years, 63.6% were males, and 39.8% had intermediate professional titles. The years of emergency work ranged from 1 to 16. The number of training courses respondents attended ranged 0 to 20, with the mean value of 3.97 ± 3.64. According to the validity and reliability analysis, Cronbach’s α coefficient was 0.975 for total questionnaire survey and ranged 0.887 and 0.975 for each questionnaire, which suggested that the scale was credible. ICC value of scale was 0.933, and the ICCs of 5 domains were 0.945 (professional quality), 0.902 (psychological resilience), 0.891 (ability to assess aftermath), 0.894 (emergency knowledge), 0.931(emergency skills), respectively. Factor analysis of the 5 domain scale showed that χ^2^ = 1001.461, *df* = 485, *p* < 0.001, *RMR* = 0.042, *IFI* = 0.920, *TLI* = 0.906, *CFI* = 0.977, *RMSEA* = 0.079, indicating a close fit.

**Table 2:** Basic information of 118 participators

Characteristics		Participators (*n* = 118)	Percentage (%)
Gender	Male	75	63.6
	Female	43	36.4
Age (year)	21–30	14	11.9
	31–40	71	60.2
	41–50	24	20.3
	51–60	9	7.6
Experience of emergency work (year)	1–3	27	22.9
	4–6	43	36.1
	7–9	21	17.8
	10–12	15	12.7
	13–15	5	4.2
	≥ 16	7	5.9
Professional titles	Senior	10	8.5
	Associate senior	27	22.8
	Intermediate	47	39.8
	Junior	33	27.9
	Worker	1	0.8
Emergency training (time)	0	23	19.4
	1	10	11.8
	2	17	14.4
	3	20	16.9
	4	13	11.0
	5	13	12.0
	6	7	5.9
	7	3	2.5
	8	1	0.9
	9	2	2.2
	10	8	7.0
	14	1	0.8
Regions	Shandong	37	31.4
	Xinjiang	32	27.1
	Sichuan	22	18.6
	Shanghai	27	22.8

**Fig. 3:**
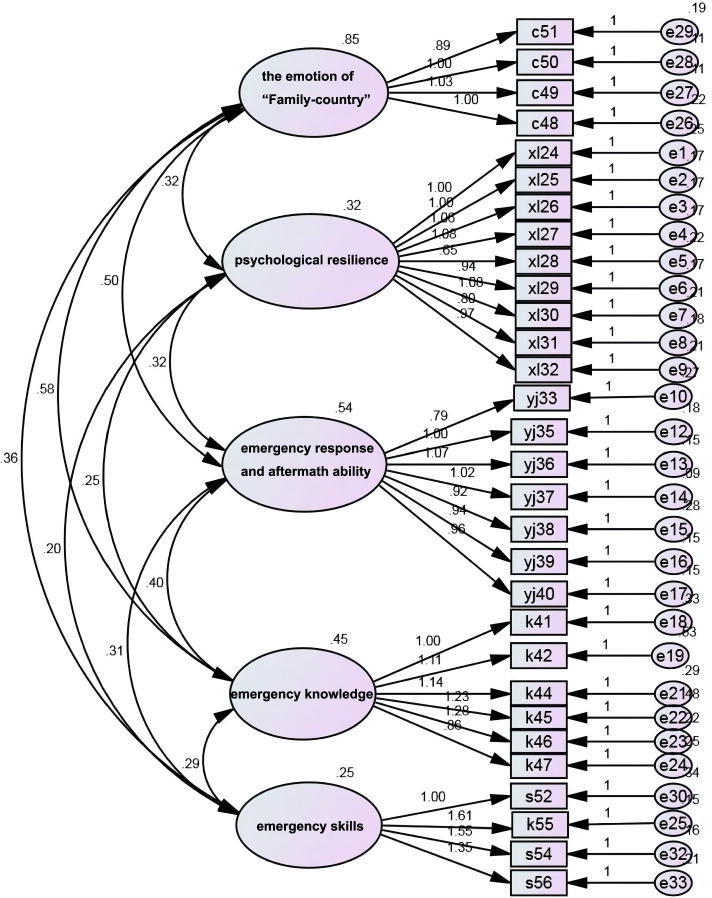
The final version of competency model for medical workers

According to the fit revision index, yj33 and yj34 items showed high similarity and the question about yj34 item was dropped. For another round of fit revision, k43 and s53 items were deleted and k55 item was merged to the domain of emergency skill. After revision, factor analysis elicited that χ^2^ = 722.4, *df* = 395, *p* < 0.001, *IFI* = 0.939, *TLI* = 0.927, *CFI* = 0.991, *RMSEA* = 0.067, which indicated a better model fit. The final version of competency model with 5 dimensions and 31 items was illustrated in Fig. 3.

### Current status of competency development of medical workers

In order to assess the current status of competency development in medical workers the emergency medical team against COVID-19, we conducted a questionnaire survey based on the final version of the competency model. Total 227 of 270 participators responded to the questionnaires effectively. As depicted in Table 3, the age of the 227 participators ranged from 21 to 60 years and the emergency work experience ranged from 1–16 years. The mean number of trainings for medical workers was 3.960 ± 3.643 (range: 0–20). There were 161 males accounting for 70.9%. In addition, subjects aged 31–40 years took account for 61.2% and those with intermediate titles accounted for 39.6%. All these suggested that the majority of members in emergency medical teams were young males. Besides, the overall Cronbach’s α coefficient was 0.975 and the sub-Cronbach’s α coefficient ranged from 0.887 to 0.975. All these indicated a good quantity of internal consistency and viability of the competency model.

**Table 3:** The basic information of 227 participators

Variables		Count	Percentage (%)
Gender	Male	161	70.9
	Female	66	29. 1
Age (year)	21–30	23	10. 1
	31–40	139	61.2
	41–50	48	21. 1
	51–60	17	7.5
Working experience (year)	1–3	57	25. 1
	4–6	71	31 3
	7–9	44	19.4
	10- 12	32	14. 1
	13- 15	9	4.0
	≥ 16	14	6.2
Professional titles	Senior	20	8 8
	Associate senior	52	22.9
	Intermediate	90	39.6
	Junior	63	27.8
	Worker	2	0.9
Emergency training (time)	0	39	17 2
	1	20	8.8
	2	25	11.0
	3	38	16.7
	4	25	11.0
	5	29	12.8
	6	14	6.2
	7	7	3.1
	8	2	0.9
	9	5	2.2
	10	16	7.0
	12	1	0.4
	14	2	0.9
	16	1	0.4
	20	3	1.3
Regions	Estern	48	21. 1
	Central	28	12.3
	Northern	39	17.2
	Southwest	19	8.4
	Northwest	30	13.2
	Southern	38	16.7
	Northeast	25	11.0

The mean scores of subscales were 4.635 ± 0.580 for professional quality, 4.367 ± 0.624 for psychological resilience, 4.0004.367 ± 0.717 for ability to assess aftermath, and 4.130 ± 0.645 for emergency skills, respectively. All the scores were more than 4, indicating that the competences in the four domains were relatively eligible. The mean score for emergency knowledge domain was 3.848 ± 0.690, suggesting the shortage of emergency knowledge in medical workers.

There were 161 male responders, more than female responders (*n* = 66), which was in line with the characteristics of health emergency work. However, there was no significant difference in the scores of 5 competency domains with regard to gender (data not shown). Notably, the scores in 5 domains were remarkably different among responders from 7 different regions (all *p* < 0.001, Table 4).

**Table 4:**
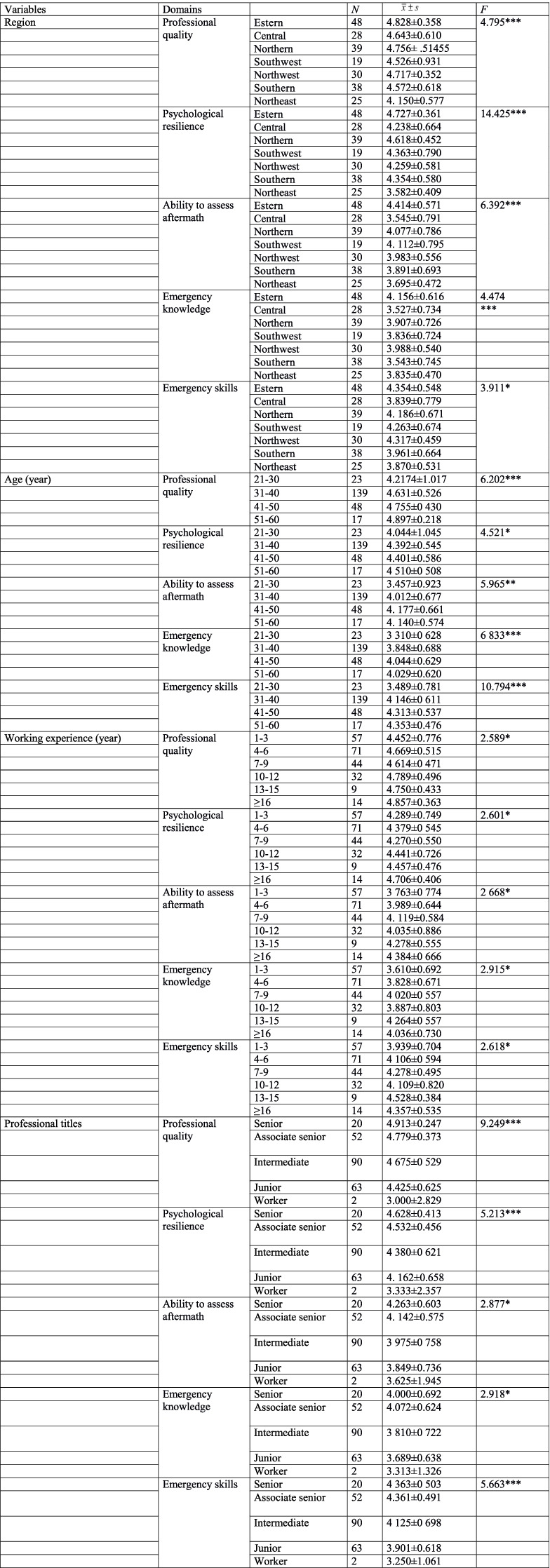
Multi-way ANOVA for the competency scores with regard to region, age, working experience, and professional title

^*^p < 0.05, **p < 0.01, ***p < 0.001

Then, the difference between any two groups were analyzed by Post hoc test. Results showed that the professional quality scores for responders from Eastern China were remarkably higher than those from Southwest China, Southern China and Northeast China (all *p* < 0.05). Additionally, the mean score in Northeast region group was significantly lower compared with north China, Southwest, Northwest region and South China group (all *p* < 0.05). All the results suggested that the performance of responders in professional quality domain from Eastern China was relative good, while the responders from northeast region exhibited poor quality. Similarly, responders in Eastern China group showed a higher level performance in psychological resilience domain than other regions (all *p* < 0.05), and those from northeast region exerted a lower level of performance (all *p* < 0.05). In the domain of aftermath assessment, responders in Eastern China presented the highest ability, followed by Northern China, Northeast region and Central China, which was similar regarding to the domain of emergency knowledge. Furthermore, Eastern China group showed the highest score in emergency skills domain, followed by northwest and North China group (Supplemental Table 1). All these suggested that medical workers in Eastern China group performed well in all dimensions of competences, while the medical workers from Northeast China had a relatively poor performance in five dimensions.

Then, we analyzed the competency level of health emergency responders at different ages, working experiences, professional titles. Results showed that there was an obvious difference in the competency scores of 5 domains among different ages (all *p* < 0.05, Table 4). As depicted in supplemental Table 2, the competency score was increased in a age-dependent manner (all *p* < 0.05). Responders aged 31–60 showed highest competency scores in 5 domains, while those aged 21–30 presented with lowest competency scores. Additionally, the competency score of medical workers was correlated with working experience (Table 4). There were significant differences in competency scores of medical workers with different length of emergency working (all *p* < 0.05). Medical workers with the years of medical working above 13 had relatively preferable competency score in 5 domains. Post hoc test revealed that the medical workers with 1–3 years of medical working showed the lowest competency scores in 5 domains. Significantly lower competency scores in the domains of ability to assess aftermath, emergency knowledge, emergency skills were observed in subjects with 1–3 years of medical working than those with medical working experience of 7–9 years, 13–15 years and above 16 years. It was worth noting that there was no significant difference in the psychological resilience score of medical staffs with 1–3 years of medical working compared with those with working experience below 15 years (Supplemental Table 3). Furthermore, we analyzed the effect of professional title on the competency score. One-way ANOVA suggested that the competency scores in 5 domains were title dependently. The medical workers with senior professional title had the highest scores, while workers without titles were present with lowest scores (Table 4). Afterwards, post hoc test revealed that medical staffs with junior and worker titles had lower competency score in both professional quality, psychological resilience and emergency skills domain than those with other three titles (all *p* < 0.05). In the ability to assess aftermath, the mean score of workers with junior title was remarkably lower than those with senior and associate senior (*p* < 0.05). Besides, differential scores related with emergency knowledge were observed between medical workers with associate senior and those with intermediate and junior titles. However, there was no significant difference in the scores of senior and worker title group compared with others (Supplemental Table 4).

### Correlation analysis

The correlations of competency score with working experience, number of training and age were analyzed by Pearson correlation analysis. Results indicated that the length of emergency work was positively correlated with the competency scores in domains of professional quality (*r* = 0.187, *p* = 0.005), psychological resilience (*r* = 0.132, *p* = 0.048), ability to assess aftermath (*r* = 0.217, *p* = 0.001), emergency knowledge (*r* = 0.205, *p* = 0.002), emergency skills (*r* = 0.191, *p* = 0.004). Thus, medical workers with long working years were correlated with high-level competences. Similarly, age was positively correlated with competency scores in 5 domains, reflected by *r* = 0.25 and *p* < 0.001 for professional quality, *r* = 0.133 and *p* = 0.045 for psychological resilience, *r* = 0.215 and *p* = 0.001 for ability to assess aftermath, *r* = 0.242 and *p* < 0.001 for emergency knowledge, *r* = 0.288 and *p* < 0.001 for emergency skills. Moreover, the number of training trials were positively correlated with the competency scores in professional quality (*r* = 0.149, *p* = 0.025), ability to assess aftermath (*r* = 0.162, *p* = 0.014), and emergency skills (*r* = 0.135, *p* = 0.042). However, no significant correlation was found between the number of training trials with the scores in psychological resilience (*r* = 0.104, *p* > 0.05) and emergency knowledge (*r* = 0.113, *p* > 0.05). Thus, significant associations were found between basic variables and competency scores, suggesting it was suitable for regression analysis.

### Regression analysis

In the present study, multiple regression analysis was conducted to evaluate the effect of 3 variables on medical worker competency (Table 5). Based on backward elimination procedure, there were three approved models for the independent variables of number of training trials, age and working experience on professional quality competency (all *p* < 0.01). The regression analysis revealed that age showed a positive association with professional quality competency reflected by β = 0.168 and *p* < 0.01 in model 1, β = 0.178, *p* < 0.001 in model 2, and β = 0.196, *p* < 0.001 in model 3. Age as an independent variable could explain 5.8% (adjusted *R*^2^ = 0.058) variance of professional quality competency. In addition, model 3 was supported for variance of psychological resilience domain. In model 3, age was the independent variable (β = 0.112, *p* < 0.05) for the psychological resilience competency and 1.3% (adjust *R*^2^ = 0.013) of variance could be explained by age. With regard to ability to assess aftermath domain, two models were approved (all *p* < 0.001). Based on model 2, age and number of training trials explained 0.5% (adjust *R*^2^ = 0.05) variance of the ability to assess aftermath. Age had the positive relationship with the ability to assess aftermath (β = 0.179, *p* < 0.001). In the multiple regression analysis of emergency knowledge as dependent variable, three regression models were significant. In the model 3, 5.4% (adjust *R*^2^ = 0.054) of variance of emergency knowledge competency could be explained by the independent variable of age. There was positive relationship between age and emergency knowledge (β = 0.226, *p* < 0.001). Furthermore, three regression models for emergency skills were supported (all *p* < 0.001). Model 2 showed the optimal model fitting (adjusted *R*^2^ = 0.055, *p* < 0.001), in which age was positively correlated with emergency skills (β = 0.237, *p* < 0.001). All these discovered that age was an independent risk factor for the medical worker competency in 5 domains.

**Table 5:** Regression analysis of independent variables with 5 competency domains

Dependent variable	Modle	Independent variable	beta, β	adjust *R2*	Significance
Professional quality	1	Constant	4.012***	0.057	5.590**
		Age	0. 168**		
		Working experience	0.009		
		Number of training trails	0.013		
	2	Constant	4.001***	0.061	8.389***
		Age	0. 178***		
		Working experience	0.014		
	3	Constant	3.996***	0.058	14.971***
		Age	0. 196***		
Psychological resilience	1	Constant	4.040*	0.012	1.885
		Age	0.067		
		Working experience	0.026		
		Number of training trails	0 011		
	2	Constant	4.005*	0.014	2.607
		Age	0.096		
		Working experience	0 013		
	3	Constant	4.001*	0.013	4.057*
		Age	0. 112*		
Ability to assess aftermath	1	Constant	3.401***	0.051	5.110***
		Age	0. 119		
		Working experience	0.054		
		Number of training trails	0.018		
	2	Constant	3.328***	0.050	6.887***
		Age	0. 179***		
		Number of training trails	0.022		
Emergency knowledge	1	Constant	3. 164***	0.052	5.099***
		Age	0. 173*		
		Working experience	0.036		
		Number of training trails	0 007		
	2	Constant	3. 169***	0.055	7.526***
		Age	0. 175*		
		Working experience	0 042		
	3	Constant	3. 112***	0.054	13.996***
		Age	0.226***		
Emergency skills	1	Constant	3.311***	0.052	5.099***
		Age	0.240***		
		Working experience	-0.002		
		Number of training trails	0.011		
	2	Constant	3.314***	0.055	7.526***
		Age	0.237***		
		Working experience	0.011		
	3	Constant	3.311***	0.054	13.996***
		Age	0.251***		

*P < 0.05, **P < 0.01, and ***P < 0.001

## Discussion

A growing number of public health emergencies in the past decade has posed threat to the public health worldwide, such as natural disaster, terrorist attacks and epidemic outbreak. To address the health emergency issues, a large number of emergency medical teams has been deployed. The preparedness and response capability of these teams has been reported to be insufficient in China. Identification of the competence of medical workers in emergency medical teams plays a critical role in improving emergency response capability. Competency based model has been widely developed for identifying the competencies of workers in medical field. Wei et al. has developed a competency model for assessing the competencies and assist the training of general practitioner in rural areas [12]. In the study of Ni et al., a competency model for family physicians was developed to improve the primary care quality and patients outcomes [13]. However, the validated tools for assessing the competences of medical workers in emergency medical teams are rare.

In this study, we constructed a competency model for health emergency teams in China to assess the competences of medical workers in health emergency teams. This tool was refined from the initial version containing 5 domains and 43 items to final version of 5 dimensions (including professional quality, psychological resilience, ability to assess aftermath, emergency knowledge, emergency skills) and 31 items. In the comparison with the competency models in other countries, there are some similarities. The previous competency models for public health works [14–16] highlighted the competences with regard to the domains of communication, professional knowledges, emergency response capacity, which was similar with our results. In addition, a recent study of the competences for public health center workers has supported that the psychological factors including disaster risk perception and the self-efficacy of disaster should be taken into consideration [15]. In our competency model, ‘psychological resilience’ domain was emphasized, which was consistent with the previous study [15].

Besides, in our study, we re-defined the domain of professional quality that was closely correlated with traditional culture and national consciousness of China. The national consciousness of individuals is one of the connotations of Chinese traditional culture, which could promote the development of the community in a positive and benign direction. The epidemic of COVID-19 was broken out at the end of December 2019 in Wuhan, China, which was declared to be the public health emergency. This disease is characterized by human-to-human transmission [17]. Up to 25 January, 75,815 cases were estimated to be affected by COVID-19 in Wuhan [18] and by the end of January, it had spread to most cities of China and at least 19 other countries [18]. To address the public health concern of COVID-19, the medical team was assembled with about 4000 medical staff from other regions of China to support Wuhan. Notably, two designated makeshift hospitals and additional mobile cabin hospitals were built to response to the epidmic of COVID-19 [19]. Patients were treated by the first-line medical workers with courage and conviction all day and all night. The dedication of medical workers contributed to the prevention and control of epidemic situation as soon as possible [20]. Patriotism was determined to be the core competence of professional quality domain by several rounds of expert discussion, which made the model with Chinese characteristics, in line with China’s national conditions, and close to the post characteristics of health emergency team members.

Furthermore, the current competency characteristics of Chinese emergency medical workers for the preparedness and response to COVID-19 were measured by a questionnaire survey based on the final version of our competency model. Our results showed that the competency scores in 4 domains were higher than 4 points, while the score of emergency knowledge related competences was 3.848. These suggested that the overall level of competencies in Chinese medical workers was relatively high and there was a shortage of emergency knowledge in medical workers, which was consistent with the study of current status of health emergency human resources in Shanghai, China [21]. This phenomenon might be due to two causes. Firstly, the current status of medical workers competency was evaluated post COVID-19 outbreak. The fight against COVID-19 pandemic improved the emergency skills, the ability to solve crisis and patriotism of medical workers. Besides, the big scoring interval for the competency domains could be a limitation.

Moreover, our results revealed that age was an independent factor affecting the competencies of medical workers. The competency scores in 5 domains were increased in an age-dependent manner. A previous study of the factors affecting emergency preparedness competency showed that the general competency score of public health inspectors aged 50–59 years was significantly higher than those aged 20–29 years [22]. A recent study of Kim et al. [23] indicated that the nursing competency for clinical practice was significantly different with regard to age of participators. The older the participators, the higher the nursing competency scores. These findings were consistent with our results. The post hoc test in our study revealed that the scores of emergency competency of medical workers in 21–30 years age group was obviously lower than those in 41–50 years age and 51–60 years age group. According to the previous study [24, 25], the clinical career could be divided into 5 stages including novice stage, advanced beginner, competent stage, proficient stage and expert. In this paper, medical workers in 21–30 years age class were in naive stage or advanced beginner stage and the main task of these staffs were to identify themselves, understand the meaning of work, and explore work preference. Thus, the competency of medical workers in 21–30 years age class was lower than other age classes.

## Conclusion

In summary, a public health emergency competency model was developed with 5 domains and 31 items in our study, and the competency model was an effective and reliable tool to assess the competences of medical workers. Moreover, the overall competencies of current medical workers in China were in a high level, except for emergency knowledge related competencies.

## Supplementary Information

**Additional file 1:** **Supplemental Table 1.** Post hoc test for independent variable of regions. **Supplemental Table 2.** Post hoc test for the different ages. **Supplemental Table 3.** Post hoc test for independent variable of working experience. **Supplemental Table 4.** Post hoc test for independent variable of professional titles

## Data Availability

The datasets used and/or analysed during the current study are available from the corresponding author on reasonable request.
